# Associations of Circulating Gut Hormone and Adipocytokine Levels with the Spectrum of Gastroesophageal Reflux Disease

**DOI:** 10.1371/journal.pone.0141410

**Published:** 2015-10-27

**Authors:** Ping-Huei Tseng, Wei-Shiung Yang, Jyh-Ming Liou, Yi-Chia Lee, Hsiu-Po Wang, Jaw-Town Lin, Ming-Shiang Wu

**Affiliations:** 1 Department of Internal Medicine, National Taiwan University Hospital, Taipei, Taiwan; 2 Graduate Institute of Clinical Medicine, College of Medicine, National Taiwan University, Taipei, Taiwan; 3 Research Center for Developmental Biology and Regenerative Medicine, National Taiwan University, Taipei, Taiwan; 4 School of Medicine, Fu Jen Catholic University, New Taipei City, Taiwan; 5 Department of Internal Medicine, E-Da Hospital, Kaohsiung, Taiwan; University Hospital Llandough, UNITED KINGDOM

## Abstract

**Objective:**

The pathogenesis of gastroesophageal reflux disease (GERD) is complex and poorly understood. We aim to investigate the association of various circulating peptide hormones with heterogenous manifestations of GERD.

**Methods:**

One hundred and four patients that had experienced typical GERD symptoms (heartburn and/or acid regurgitation) for at least 3 episodes per week in the past 3 months were enrolled. All patients received a baseline assessment of symptom severity and frequency with the Reflux Disease Questionnaire and an upper endoscopy to classify GERD into erosive esophagitis (EE, n = 67), non-erosive esophagitis (NE, n = 37), and Barrett’s esophagus (BE, n = 8). Fifty asymptomatic subjects with an endoscopically normal esophagus were recruited as the control group. Complete anthropometric measures and blood biochemistry were obtained and fasting serum levels of adipocytokines (adiponectin and leptin) and gut hormones (ghrelin and peptide YY (PYY)) were determined by enzyme-linked immunosorbent assay in all subjects.

**Results:**

All circulating peptide hormone levels were not statistically different between the GERD and control groups. However, GERD patients appeared to have lower PYY levels [median (25^th^-75^th^ percentile), 80.1 (49.8–108.3) vs. 99.4 (65.8–131.9) pg/ml, *p* = 0.057] compared with control subjects. Among the GERD patients, ghrelin levels were inversely associated with the frequency and severity of acid regurgitation. In male GERD patients, EE was associated with significantly higher PYY levels [107.0 (55.0–120.8) vs. 32.8 (28.7–84.5) pg/ml, *p* = 0.026] but lower adiponectin levels [6.7 (5.6–9.3) vs. 9.9 (9.6–10.6) μg/ml, *p* = 0.034] than NE. Patients with BE had significantly lower adiponectin levels [6.0 (5.1–9.2) vs. 9.2 (7.1–11.2) μg/ml, *p* = 0.026] than those without BE.

**Conclusions:**

Humoral derangement of circulating peptide hormones might participate in inflammation and symptom perception in patients suffering from GERD. Further studies to clarify the exact role of these hormones in the pathogenesis of GERD are warranted.

## Introduction

Gastroesophageal reflux disease (GERD) has been associated with a broad spectrum of symptoms, including typical symptoms, such as heartburn and regurgitation, and several extra-esophageal symptoms, such as laryngitis and coughing, all of which greatly impact quality of life [1]. GERD can be further stratified into erosive esophagitis (EE) and its non-erosive (NE) counterpart based on the results of endoscopic evaluation. Long-term GERD has also been associated with the development of Barrett’s esophagus (BE) and esophageal adenocarcinoma [2].

The incidence and prevalence of GERD have increased remarkably over the past few decades in Taiwan and the rest of the world [3]. The pathogenesis of GERD is multi-factorial and is not fully understood. Another significant health problem with a similar increasing trend of prevalence in Western countries is obesity [4]. Previous epidemiologic studies have found associations of obesity (in terms of high body mass index (BMI)) with GERD symptoms, EE, and BE [3, 5–7]. In addition, visceral obesity has also been found to be an important risk factor for reflux esophagitis in cross-sectional studies [8–10]. The exact pathophysiology that links obesity and visceral obesity with GERD has not been fully elucidated. Changes in gastroesophageal anatomy and physiology due to obesity have been proposed to explain the association, which include reduced lower esophageal sphincter (LES) pressure, increased frequency of transient LES relaxation, increased prevalence of hiatal hernia and esophageal motor disorders, elevated intragastric pressure, and disorders of gastric accommodations [11]. Several peptide hormones secreted by the adipose tissue and the gut play an important role in regulating food intake, gastrointestinal motility, and energy balance. The answer to whether these humoral factors contribute to the development and progression of GERD remains elusive. We hypothesized that these humoral factors, including various adipocytokines and gut hormones, may participate in the pathogenesis of GERD and its related complication of BE. In this study, therefore, we first compared the circulating levels of index gut hormones (ghrelin and peptide YY (PYY)) and adipocytokines (adiponectin and leptin) in GERD patients and normal control subjects. We also explored the relationship between the circulating levels of these peptide hormones and the spectrum of GERD (i.e., NE, EE, and BE) as well as the symptom profile in GERD patients.

## Material and Methods

### Ethics Statement

This study was approved by the Ethical Committee of National Taiwan University Hospital prior to implementation and all subjects provided written informed consent.

### Study population

Consecutive patients with symptoms suggestive of GERD were enrolled from gastroenterology outpatient clinics. Typical GERD symptom(s) were defined as heartburn and/or acid regurgitation experienced for at least 3 episodes per week for a minimum of 3 months. Those who received concurrent proton pump inhibitor (PPI) treatment, reported a history of peptic ulcer disease or gastrointestinal surgery, peptic ulcer disease or malignancy proven by endoscopy, or were unwilling or unable to provide informed consent were excluded from the study. Asymptomatic subjects who received endoscopy for cancer screening purposes and had an endoscopically normal esophagus were recruited as the control group.

### Questionnaire and Anthropometry

All subjects were interviewed by well-trained research nurses using a structured questionnaire to obtain information on demographic variables. In addition, they were asked to complete a validated questionnaire, the Reflux Disease Questionnaire (RDQ), to evaluate their gastroesophageal reflux symptoms [12]. The RDQ comprises 12 questions assessing the frequency and severity of heartburn, regurgitation, and dyspepsia on 3 subscales. All questions were scored on a Likert scale with scores ranging from 0 to 5 for frequency (‘not present’ to ‘daily’) and severity (‘not present’ to ‘severe’). The presence of each related symptom was verified by a gastroenterologist (P.H.T.). Anthropometric measurements were performed by certified nurses. BMI (kg/m^2^) was calculated by dividing body weight (kg) by squared height (m^2^).

### Endoscopy

After an overnight fast, subjects were placed in the left lateral decubitus position. Endoscopy was performed by an experienced endoscopist (M.H.W.). During the procedure, the stomach and duodenum were inspected to exclude possible lesions. A biopsy was performed in the stomach to verify *Helicobacter pylori* infection status. The definition of EE rests on the demonstration of diffuse or streaking erythema, mucosal friability, erosions, or ulcers, *i*.*e*., mucosal breaks. With this standard, the distal portion of the esophagus was evaluated carefully to determine the presence of any mucosal injury. The severity of EE was assessed according to the Los Angeles (LA) classification with standard comparator photos [13]. BE was suspected when a tongue-like extension of salmon-colored mucosa from the esophagogastric junction (EGJ, defined as where the proximal end of the gastric folds meet the tubular esophagus) was identified, and was confirmed by histological identification of specialized columnar epithelia with intestinal metaplasia. Hiatal hernia was defined as a distance of at least 2 cm between the EGJ and the diaphragmatic hiatus.

### Blood biochemistry and peptide hormone assay

A fasting blood sample was taken on the day of endoscopy. The blood biochemistry, including the blood glucose, total cholesterol, and triglyceride levels, was measured with both internal and external quality control procedures accredited by the College of American Pathologists. Blood samples for the measurement of peptide hormones were stored on ice during collection and then immediately centrifuged. Serum was separated and stored at -80°C until assay. Serum concentrations of total ghrelin (EMD Millipore Corporation, Missouri, USA), PYY (EMD Millipore Corporation), leptin (B-Bridge International, Inc., Cupertino, CA, USA) and adiponectin (B-Bridge International, Inc.) were analyzed in duplicate using commercial enzyme-linked immunosorbent assay kits, according to the manufacturer’s instructions. The intra-assay and inter-assay variabilities were 1.3% and 7.8% for ghrelin, 2.7% and 6.9% for PYY, 4.2% and 6.7% for leptin, and 4.6% and 3.3% for adiponectin, respectively.

### Statistical analysis

Kolmogorov-Smirnov tests were used to test for normal distribution of various variables. Continuous data were expressed as mean ± standard deviation (SD) for normal distributions and compared by a Student’s *t*-test. For skewed variables, which included all peptide hormone levels, data were expressed as median (25^th^-75^th^ percentile) and compared with Wilcoxon two-sample test. Categorical data were expressed as percentage and analyzed by Pearson χ^2^ tests or Fisher exact tests, as appropriate. Ordinal logistic regressions were performed to assess the associations between respective peptide hormone levels and symptom frequency/severity scores of each RDQ item. After the scale of each peptide hormones was divided by a factor of 10, odds ratios (ORs) with 95% confidence intervals (CIs) between each peptide hormone and each questionnaire item score were calculated. Furthermore, univariable relationships between respective peptide hormone levels and various risk factors of GERD were assessed with Spearman's correlation. We also created multivariable regression models to control for possible confounders. Factors for adjustment were chosen a priori based on published risk factors for GERD, including age, gender, smoking, alcohol drinking, BMI, fasting blood glucose, triglyceride and cholesterol levels. A *p*-value of less than 0.05 was considered to indicate a statistically significant difference. All statistical analyses were performed using SPSS 16 (SPSS, Inc., Chicago, IL, USA).

## Results

### Demographics

As shown in Table 1, a total of 104 GERD patients and 50 control subjects were recruited into the study. Among the GERD group, 67 (64.4%) and 37 (35.6%) patients were classified as EE and NE, respectively. EE patients were predominantly mild in severity (49 subjects with grade A or B, 18 with grade C or D). Eight (7.7%) patients were diagnosed with BE. The average ages of all GERD patients and control group were similar: 45.6 ± 12.8 and 44.4 ± 12.9 years, respectively. A higher percentage of the GERD group was female (70.2%). No anthropometric or blood biochemical parameters were statistically different between these two groups.

**Table 1:** Demographics of gastroesophageal reflux disease (GERD) patients and control subjects.

	GERD (n = 104)	Control (n = 50)	*P* *
Age, yr	45.6 ± 12.8	44.4 ± 12.9	0.584
Male gender, n (%)	31 (29.8%)	25 (50.0%)	0.02*
BMI, kg/m^2^	22.4 ± 3.0	22.8 ± 2.6	0.397
Abdominal girth, cm	79.9 ± 9.9	83.7 ± 11.4	0.093
Hip girth, cm	94.7 ± 7.1	97.7 ± 10.3	0.096
*H*. *pylori* infection, n (%)	28 (26.9%)	0	-
Fasting blood glucose, mg/dL	84.9 ± 11.9	89.2 ± 19.2	0.145
Triglycerides, mg/dL	106.9 ± 58.9	119.6 ± 62.1	0.132
Total cholesterol, mg/dL	205.5 ± 43.8	216.9 ± 43.3	0.223
***Peptide hormones***			
Ghrelin, pg/mL	162.4 (82.0–240.4)	160.5 (81.8–258.9)	0.647
PYY, pg/ml	80.1 (49.8–108.3)	99.4 (65.8–131.9)	0.057
Adiponectin, μg/mL	9.0 (6.8–11.1)	7.7 (6.3–9.5)	0.087
Leptin, ng/mL	8.4 (5.1–12.8)	7.7 (4.4–13.3)	0.427
***RDQ scores***	21.7 ± 10.9	-	
***Endoscopic findings***			
Hiatal hernia, n (%)	1 (0.1)	-	
Barrett’s esophagus, n (%)	8 (7.7)	-	
LA Grade A+B, n (%)	49 (47.1)	-	
LA Grade C+D, n (%)	18 (17.3)	-	
Negative, n (%)	37 (35.6)		

Data are presented as mean ± standard deviation, median (25^th^-75^th^ percentile), or number (percentage), as appropriate.

Abbreviations: BMI, body mass index; RDQ, Reflux Disease Questionnaire; LA, Los Angeles classification system; PYY, peptide YY.

*P < 0.05, indicates statistical significance.

### Circulating levels of peptide hormones in GERD patients and control subjects

As shown in Table 1, all four circulating peptide hormone levels were not statistically different between the GERD and control groups. However, GERD patients appeared to have lower PYY levels [80.1 (49.8–108.3) vs. 99.4 (65.8–131.9) pg/ml, *p* = 0.057] compared with control subjects. We further compared anthropometric parameters, blood biochemistry, and circulating peptide hormone levels in EE and NE patients (Table 2). Compared with NE patients, EE patients had a larger abdominal and hip girth than NE patients (81.5 ± 10.2 vs. 77.1 ± 8.7 cm, *p* = 0.031; 95.7 ± 6.9 vs. 92.8 ± 7.2 cm, *p* = 0.044). No circulating peptide levels were significantly different between EE and NE patients. However, in male patients, EE was associated with a significantly larger abdominal girth (84.7 ± 11.6 vs. 76.2 ± 6.2 cm, *p* = 0.016), higher triglyceride levels (129.8 ± 55.8 vs. 93.1 ± 32.0, *p* = 0.034), and higher PYY levels [107.0 (55.0–120.8) vs. 32.8 (28.7–84.5) pg/ml, *p* = 0.026] but lower adiponectin levels [6.7 (5.6–9.3) vs. 9.9 (9.6–10.6) μg/ml, *p* = 0.034 [than NE. The anthropometric parameters, blood biochemistry, and circulating peptide hormone levels were not significantly different between female EE and NE patients. Patients with BE had significantly lower adiponectin levels [6.0 (5.1–9.2) vs. 9.2 (7.1–11.2) μg/ml, *p* = 0.026] than those without BE.

**Table 2:** Comparison of anthropometric parameters, blood biochemistry, endoscopic findings, and peptide hormone levels between erosive esophagitis (EE) and non-erosive reflux disease (NE) patients.

	All			Male			Female		
	EE (n = 67)	NE (n = 37)	*P* *	EE (n = 23)	NE (n = 8)	*P* *	EE (n = 44)	NE (n = 29)	*P* *
Age, yr	45.9 ± 12.9	45.0 ± 12.7	0.740	43.1 ± 10.8	42.6 ± 11.7	0.914	47.3 ± 13.7	45.7 ± 13.1	0.607
BMI, kg/m^2^	22.4 ± 2.9	22.3 ± 3.3	0.838	22.9 ± 3.8	22.3 ± 4.0	0.714	22.1 ± 2.2	22.3 ± 3.2	0.854
Abdominal girth, cm	81.5 ± 10.2	77.1 ± 8.7	0.031*	84.7 ± 11.6	76.2 ± 6.2	0.016*	79.7 ± 9.1	77.3 ± 9.4	0.273
Hip girth, cm	95.7 ± 6.9	92.8 ± 7.2	0.044*	98.1 ± 8.0	93.2 ± 7.4	0.138	94.5 ± 5.8	92.7 ± 7.2	0.254
Fasting blood glucose, mg/dL	85.4 ± 12.1	83.9 ± 11.6	0.549	87.7 ± 12.3	84.9 ± 10.1	0.566	84.2 ± 12.0	83.7 ± 12.1	0.853
Triglycerides, mg/dL	111.2 ± 52.0	99.3 ± 69.7	0.310	129.8 ± 55.8	93.1 ± 32.0	0.034*	101.4 ± 47.6	101.1 ± 77.4	0.977
Total cholesterol, mg/dL	208.8 ± 44.8	199.6 ± 41.8	0.327	213.0 ± 52.5	201.4 ± 64.0	0.612	206.6 ± 40.8	199.2 ± 35.0	0.426
Ghrelin, pg/mL	164.2 (83.8–241.6)	158.1 (73.6–212.4)	0.864	86.2 (55.8–221.9)	149.2 (74.5–195.1)	0.495	178.2 (108.4–242.9)	162.5 (70.8–246.9)	0.281
PYY, pg/ml	81.2 (50.9–111.2)	77.3 (39.5–104.2)	0.683	107.0 (55.0–120.8)	32.8 (28.7–84.5)	0.026*	76.3 (49.8–107.0)	85.6 (60.3–116.4)	0.480
Adiponectin, μg/mL	8.5 (6.6–10.9)	10.0 (7.4–11.6)	0.177	6.7 (5.6–9.3)	9.9 (9.6–10.6)	0.034*	9.0 (7.7–11.7)	10.0 (7.0–12.0)	0.960
Leptin, ng/mL	8.5 (5.1–12.3)	8.4 (4.9–13.7)	0.874	5.0 (3.6–7.4)	3.0 (1.1–10.1)	0.121	9.9 (7.6–15.6)	8.7 (7.4–15.7)	0.626

Data are presented as mean ± standard deviation or median (25^th^-75^th^ percentile), as appropriate.Abbreviations: BMI, body mass index; PYY, peptide YY.

*P < 0.05, indicates statistical significance.

### Association of peptide hormone levels with Reflux Disease Questionnaire scores in GERD patients

As shown in Table 3, an increase in ghrelin level (expressed in 10 pg/mL) was associated with an decrease in the odds of the frequency and severity of an acid taste in the mouth, (OR = 0.964, 95% CI = 0.933–0.995, *p* = 0.024; OR = 0.969, 95% CI = 0.939–1, *p* = 0.047, respectfully); an increase in adiponectin level (expressed in 10 μg/mL) was associated with an lower risk of the severity of burning in the upper stomach (OR = 0.349, 95% CI = 0.127–0.957, *p* = 0.041).

**Table 3:** Association of peptide hormone levels (divided by 10) with respective symptom scores of Reflux Disease Questionnaire in patients with gastroesophageal reflux disease based on ordinal regression models.

	Ghrelin	PYY	Adiponectin	Leptin
**Burning behind the breast bone**				
Frequency	0.975 (0.941–1.009)	1.007 (0.938–1.081)	0.638 (0.22–1.846)	1.004 (0.702–1.435)
Severity	0.978 (0.946–1.012)	1.054 (0.979–1.134)	0.634 (0.229–1.698)	1.031 (0.711–1.495)
**Pain behind the breast bone**				
Frequency	1.023 (0.992–1.055)	0.993 (0.926–1.065)	0.752 (0.281–2.016)	1.041 (0.691–1.568)
Severity	1.022 (0.991–1.054)	0.996 (0.925–1.072)	0.760 (0.284–2.033)	1.104 (0.718–1.697)
**Burning in the upper stomach**				
Frequency	0.981 (0.95–1.014)	1.036 (0.964–1.114)	0.443 (0.165–1.189)	0.987 (0.684–1.425)
Severity	0.986 (0.955–1.018)	1.039 (0.969–1.114)	0.349 (0.127–0.957)*	0.943 (0.645–1.379)
**Pain in the upper stomach**				
Frequency	1.003 (0.972–1.034)	0.998 (0.922–1.079)	0.690 (0.265–1.797)	1.018 (0.711–1.457)
Severity	1.003 (0.973–1.034)	0.997 (0.921–1.078)	0.554 (0.207–1.478)	1.020 (0.693–1.500)
**Acid taste in the mouth**				
Frequency	0.964 (0.933–0.995)*	0.960 (0.892–1.034)	0.590 (0.216–1.608)	1.025 (0.704–1.491)
Severity	0.969 (0.939–1)*	0.976 (0.9–1.059)	0.446 (0.17–1.171)	1.237 (0.838–1.826)
**Movement of materials upwards from the stomach**				
Frequency	1.001 (0.971–1.031)	1.034 (0.964–1.11)	2.373 (0.924–6.096)	0.828 (0.565–1.215)
Severity	1.008 (0.978–1.039)	1.045 (0.97–1.126)	1.270 (0.529–3.05)	0.859 (0.582–1.269)

Data are presented as odds ratio (95% confidence interval)

* *P*-value < 0.05

### Factors associated with various peptide hormone levels

As shown in Table 4, univariate and multivariate analyses showed that these peptide hormone levels were associated with several risk factors of GERD, including male gender, blood metabolic profile, and markers of general and central obesity. While BMI was independently associated with circulating levels of ghrelin (*p* = 0.019) and adiponectin (*p* = 0.016), abdominal girth and hip girth were independently associated with those of leptin (both *p*<0.001). Fasting blood glucose and triglyceride were independently associated with ghrelin and adiponectin levels, respectively (*p*<0.001 and 0.002, respectively). Male gender was independently associated with lower levels of both leptin (*p*<0.001) and adiponectin (*p* = 0.002).

**Table 4:** Factors associated with respective peptide hormone levels.

	Correlation		Multivariable regression		
	r	*P* *	β	SE	*P* *
**Ghrelin**					
BMI	-0.244	0.008	-10.312	4.346	0.019
Abdominal girth	-0.201	0.043	-0.084	1.660	0.960
Fasting blood glucose	0.447	<0.001	5.097	0.897	<0.001
**Leptin**					
Age	0.195	0.015	0.007	0.065	0.919
Male gender	-0.374	<0.001	-8.710	1.676	<0.001
Abdominal girth	0.334	<0.001	0.402	0.074	<0.001
Hip girth	0.348	<0.001	0.468	0.094	<0.001
Fasting blood glucose	0.205	0.011	0.165	0.053	0.002
**Adiponectin**					
Male gender	-0.340	<0.001	-2.195	0.685	0.002
BMI	-0.253	0.002	-0.257	0.106	0.016
Abdominal girth	-0.344	<0.001	-0.023	0.036	0.519
Hip girth	-0.241	0.007	-0.018	0.044	0.683
Triglyceride	-0.373	<0.001	-0.020	0.006	0.001

Multivariable regression analysis was performed adjusting for common risk factors of GERD, including age, gender, smoking, drinking, BMI, fasting blood glucose, triglyceride and cholesterol levels.Abbreviations: BMI, body mass index; GERD, gastroesophageal reflux disease

*P < 0.05 indicates statistical significance.

## Discussion

The present study demonstrated that the derangement of various circulating peptide hormones might be a contributing factor to the mucosal inflammation and symptom perception of GERD. Compared with control subjects, GERD patients appeared to have lower PYY levels, although this did not reach statistical significance. EE was associated with significantly higher PYY levels but lower adiponectin levels than NE in male patients. Fasting ghrelin levels were negatively associated with the frequency of an acidic taste in the mouth based on the RDQ scores.

Among the various gut hormones, PYY is released from L-cells of the distal gut into the circulation. PYY increases satiety, reduces food intake, delays gastric emptying, and regulates energy expenditure, and has been associated with several GI diseases, including irritable bowel syndrome, inflammatory bowel disease, celiac disease, and diabetic gastroenteropathy [14]. In the present study, GERD patients seemed to have lower PYY levels, which was consistent with another study conducted by Perdikis *et al*. In their study, basal levels of PYY were moderately decreased in 20 GERD patients compared with 8 control subjects irrespective of LES pressure [15]. However, the diagnosis of GERD was based on 24-h ambulatory pH monitoring and no further endoscopic classification was performed. In the present study, although PYY levels were not significantly different between EE and NE patients, NE was associated with lower PYY levels than EE in male patients but not female patients. The reason for the gender difference of PYY on GERD manifestation remains unclear. Masaka *et al*. have found a prominent gender difference in the severity of esophageal tissue damage in a GERD animal model and suggested the critical involvement of estrogen in controlling GERD-related esophageal epithelial injury [16]. However, in a recent human study conducted by Menon *et al*., female sex hormone levels did not appear to contribute to GERD once adjustment was made for the influence of increasing BMI [17]. Although in the present study there was no significant correlation between PYY levels and respective GERD symptoms, whether the changes of PYY in the GERD spectrum reflect an etiological factor or an adaptive response to pathophysiological alterations deserves further investigation.

Ghrelin, an endogenous ligand of a growth hormone (GH) secretagogue receptor and a potent GH-releasing agent, acts as an orexigenic gut hormone secreted by the gastric mucosa in response to hunger. Circulating ghrelin levels vary widely in diverse conditions of the upper gastrointestinal tract, reflecting the inflammatory and atrophic events of the gastric mucosa [18]. Ghrelin stimulates GI motility and gastric emptying and thus has been implicated as a pathogenetic factor in GI motility disorders such as gastroparesis and functional dyspepsia. Shindo *et al*. studied 151 patients with functional dyspepsia or nonerosive reflux disease and found that acylated ghrelin levels were significantly lower in NERD patients and that 33% of NERD patients exhibited delayed gastric emptying [19]. Nevertheless, another study has shown similar plasma ghrelin levels between EE patients and normal controls [18]. In the present study, fasting total ghrelin levels were not significantly different between the GERD and control groups. Ghrelin levels were also similar between EE and NE patients. However, fasting ghrelin levels inversely associated with the symptom frequency and severity of “acid taste in the mouth” based on the RDQ. In addition, ghrelin levels appeared to be lower in BE patients compared with EE and NE patients, but the difference was not statistically significant (*p* = 0.117), probably due to the small number of BE patients. This finding was consistent with a previous study which found an inverse association of ghrelin levels and incidence of esophageal adenocarcinoma among overweight subjects [20]. Nevertheless, in a recent case-control study involving subjects undergoing colorectal screening and subjects with clinically confirmed BE, the authors found an inverse association of ghrelin with GERD symptom severity and EE but a positive association of ghrelin with BE [21]. Reasons for such conflicting results remain unclear. Since ghrelin promotes gastric emptying, lower circulating ghrelin levels may cause delayed gastric emptying and thus increase the chances acid reflux and initiate the damage-and-repair sequences of the lower esophageal mucosa. Previous studies have showed a therapeutic role of ghrelin and related compounds in functional dyspepsia, diabetic gastroparesis, and postoperative ileus after partial colectomy [18, 22, 23]. The questions of whether ghrelin plays an important role in the pathogenesis of GERD and its associated complications of BE and esophageal adenocarcinoma and whether similar therapeutic approaches with a selective ghrelin receptor agonist could be applied in the management of GERD deserves further investigation.

Adiponectin is one of the hormones secreted by adipose tissue. The correlation between low circulating adiponectin and obesity (especially central obesity), insulin resistance, and type 2 diabetes has been well established [24]. In addition, adiponectin acts on monocytes in an anti-inflammatory manner and its deficiency has been associated with several inflammatory gastrointestinal diseases, such as non-alcoholic steatohepatitis, acute pancreatitis, and inflammatory bowel diseases [25, 26]. Whether adiponectin contributes to the inflammation and/or metaplasia of the esophageal mucosa during the progression of GERD remains elusive.

In the present study, EE was associated with significantly lower adiponectin levels than NE in male patients, which is consistent with a recent study by Kato *et al*. [27]. The authors analyzed 2405 subjects who underwent a health check-up and found that men with low serum adiponectin levels were more susceptible to EE. However, they did not investigate the association of adiponectin with GERD symptoms. Our study has further shown that adiponectin levels were also inversely associated with severity of burning in the upper stomach, which was consistent with a Japanese study showing an inverse relationship between adiponectin levels and GERD symptoms in obese patients [28]. Hirata *et al*. also showed that coexistence of metabolic syndrome and low levels of serum adiponectin was associated with a higher prevalence and higher frequency of GERD symptoms in subjects with type 2 diabetes mellitus [29]. A recent study further demonstrated that visceral fat may increase the risk of reflux esophagitis by increasing the levels of inflammatory cytokines [30]. The underlying mechanism for the inflammation and symptom perception may be partially explained by the anti-inflammatory and neurosensorial protective effect of adiponectin, as shown in our previous studies [31, 32]. Moreover, significantly lower plasma adiponectin levels were noted in BE patients in the present study, which are similar results to those of a case-control study conducted by Rubensein *et al*. [33]. Despite methodologic limitations and the small sample size (50 subjects in each group), the authors suggested that the effects of abdominal obesity on the risk of BE may be mediated by adiponectin and other circulating factors, rather than simply a mechanical effect of obesity promoting GERD. In a recent case—control study, BE was associated with circulating inflammatory cytokines, leptin, low levels of anti-inflammatory cytokines and statistically non-significant lower adiponectin levels, which also supports the role of inflammation in the development of BE [34]. In another study investigating the role of adiponectin in the early rise in the postprandial glycemic response, Iwase *et al*. showed that delayed gastric emptying was associated with lower high-molecular weight (HMW) adiponectin, but not total adiponectin [35]. Although HMW adiponectin was not measured in the present study, further studies with a HMW adiponectin assay may help to clarify the pathophysiology of GERD. Taken together, these findings suggest that decreased anti-inflammatory cytokines, such as adiponectin, may be associated with the development of GERD-related reflux symptoms, EE, and BE.

Leptin, also a major product of adipose tissue and the GI tract, signals nutritional status to the central nervous system and peripheral organs [36]. Leptin also has proliferative and anti-apoptotic effects [37]. Higher circulating leptin levels have been associated with an increased risk of BE in most previous studies [21, 34, 38, 39]. Moreover, Leptin has also been found to have a positive association with reflux esophagitis that was independent of visceral fat [30]. In the present study, however, circulating leptin levels were associated with gender and abdominal and hip girth, but not the GERD spectrum and symptomatology: our findings are consistent with those of a histopathological study by Francois *et al*. [40]. Further studies may be needed to elucidate the role of leptin linking metabolic syndrome, obesity, and GERD.

With the help of FDG PET/CT, we demonstrated in a previous study that obesity markers, both visceral and general, are independent determinants of esophageal inflammation in GERD patients [10]. In theory, central obesity raises intragastric pressure and thus predisposes to esophageal acid exposure. Moreover, visceral fat is biologically active and produces a variety of factors, including IL-6 and TNF-α, which may affect esophageal mucosa integrity and inflammation. In the present study, BMI was independently associated with lower ghrelin and adiponectin levels while abdominal girth was associated with a higher leptin level, consistent with previous studies.[31, 41]. These findings suggest that various gut hormones might play pivotal roles in the complex relationship between obesity and GERD.

The present study has several limitations. First, the sample size was relatively small and the number of patients in each GERD spectrum disease was limited, especially those with BE. A lack of sufficient statistical power to demonstrate differences between each disease group could have led to type II errors. Second, two forms of ghrelin, the acylated and desacyl forms, circulate in the blood. However, only total ghrelin levels were measured. Although total ghrelin levels correlate well with acylated ghrelin levels, further studies measuring both forms of ghrelin may help to explore the role of ghrelin in the pathophysiology and management of GERD. Third, functional studies of GERD, like 24-h ambulatory pH or impedance-pH monitoring, were not used in the present study, and NE patients may have encompassed those with functional heartburn. Such misclassification may lead to the insignificant differences of various hormone levels between the GERD and the control group. However, all cases had undergone a comprehensive endoscopic examination and interview with a validated GERD symptom questionnaire. Future studies incorporating these functional studies, like impedance-pH monitoring, may be complementary in evaluating the complex interplay between circulating hormones and the GERD spectrum. Moreover, gastric emptying tests were not performed in the present study. Delayed gastric emptying has been implicated in the pathophysiology of GERD and its severity may be affected by the circulating peptide hormones such as ghrelin and PYY. Future studies including these gastric emptying parameters may help to clarify the roles of these various peptide hormones in the heterogenous manifestations of GERD. Finally, since this study was cross-sectional in design, the causal link between the function of the respective hormones and the spectrum of GERD could not be readily established. Further longitudinal or interventional study may help to clarify this important issue.

In conclusion, this present study highlights that humoral derangement of various circulating gut hormones and adipocytokines might participate in the erosive mucosal changes and symptom perception of GERD through different pathophysiologic processes. Further prospective studies involving a larger case number, standard acid suppressive therapy, and complete outcome measures are warranted to clarify the complex relationship of these peptide hormones with the heterogenous manifestations of GERD and to explore their therapeutic potential in the treatment of GERD.
